# Level of autistic traits in neurotypical adults predicts kinematic idiosyncrasies in their biological movements

**DOI:** 10.3389/fnint.2024.1364249

**Published:** 2024-04-24

**Authors:** Gregory F. Lewis, Elizabeth B. daSilva, Mohammad Aghajani, Surabhi Date, Mark Jaime

**Affiliations:** ^1^Socioneural Physiology Lab, Kinsey Institute, Luddy School of Informatics, Computing, and Engineering, Indiana University, Bloomington, IN, United States; ^2^Social Neuroscience Lab, Division of Science, Indiana University, Columbus, IN, United States; ^3^Department of Kinesiology, School of Public Health, Indiana University, Bloomington, IN, United States

**Keywords:** broad autism phenotype (BAP), kinematics, autistic traits, Autism Spectrum Quotient (AQ), micromovements, movement kinematics, broader autism phenotype, movement production

## Abstract

**Introduction:**

Over the last decade of research, a notable connection between autism spectrum disorder (ASD) and unique motor system characteristics has been identified, which may influence social communication through distinct movement patterns. In this study, we investigated the potential for features of the broader autism phenotype to account for kinematic idiosyncrasies in social movements expressed by neurotypical individuals.

**Methods:**

Fifty-eight participants provided recordings of point-light displays expressing three basic emotions and completed the Autism Spectrum Quotient (AQ). We extracted kinematic metrics from the biological movements using computer vision and applied linear mixed-effects modeling to analyze the relationship between these kinematic metrics and AQ scores.

**Results:**

Our results revealed that individual differences in the total AQ scores, and the sub-scale scores, significantly predicted variations in kinematic metrics representing order, volume, and magnitude.

**Discussion:**

The results of this study suggest that autistic traits may intricately influence the movement expressions at the microlevel, highlighting the need for a more nuanced understanding of the potential endophenotypic characteristics associated with social movements in neurotypical individuals.

## 1 Introduction

From early in development human social communication is centered around movements (Meltzoff and Marshall, 2018). Seminal research has demonstrated that a caregiver who abruptly stops moving during a social exchange with his or her infant can evoke anxiety in the infant (Tronick et al., 1978; Adamson and Frick, 2003). And several theoretical conceptions have posited that the coordinated exchange of movements with others is a nexus for the emergence of human social understanding (Meltzoff and Gopnik, 1993; Barresi and Moore, 1996; Moore, 1996; Gopnik and Meltzoff, 1994; Mundy, 2016). At the neurocellular level, perceptual-motor neurons serve as effectors of movement information. They have come to be known as mirror neurons. Their discovery in both nonhuman and human primates has expanded our understanding of the cortex as a resonance system, activated by the movements of others (Di Pellegrino et al., 1992; Gallese et al., 1996; Iacoboni et al., 1999). During the decades that have followed the discovery of mirror neurons, a large body of literature has emerged on the role of mirror neurons in social processes, including imitation, empathy, and even sociopathy, as well as the development of social functioning in autism spectrum disorder (ASD) (Iacoboni, 2009). Despite skepticism in the extent to which mirror neurons play a role in human social understanding, and in ASD (e.g., Hickok, 2009; Hamilton, 2013; Heyes and Catmur, 2022), mirror neuron research has shed light on the fact that movement is a fundamental source of information for social neurocognitive systems (Cook, 2016).

Movement differences permeate across the autism spectrum. Although descriptions of idiosyncratic movements in autistic children date back to the writings of Kanner, 1971 and Asperger (1944), interest in understanding the links between movement and ASD has burgeoned in the recent decade. A significant proportion of adults and children diagnosed with ASD present with differences in gross motor movements (Bhat, 2020; Bhat, 2021; Wang et al., 2022; Bhat et al., 2023), gait (Hallett et al., 1993; Esposito et al., 2011; Nobile et al., 2011; Weiss et al., 2013), the planning, control, and execution of goal-directed movements (Hughes, 1996; Glazebrook et al., 2006, 2009; Stoit et al., 2013), and fine motor movements (Beversdorf et al., 2001; Fuentes et al., 2009; Fournier et al., 2010; Johnson et al., 2013; Alaniz et al., 2015). Movement differences have also been associated with social, emotional, and behavioral disturbances (Freitag et al., 2007; Hilton et al., 2007; Sipes et al., 2011; Papadopoulos et al., 2012; Wang et al., 2022). Moreover, given that both action and perceptual feedback are integral for the accurate production of movements, it is likely that the motoric differences in individuals with ASD also alter their interpretation of social movements. The mechanism for such an impairment is posited to occur via a mismatch between the perception of movements and the observer’s internal representation of those movements (Aransih and Edison, 2019). For example, Gowen has proposed that many of the observed deficits in imitation in autism can be attributed to autistic children and adults failing to attend to relevant kinematic information that provides cues regarding intentionality and goals (Gowen, 2012). Because movement is nested in social behavior, and social impairment is a cardinal feature of autism, children with autism may not have learned the same nuanced social strategies that neurotypicals use such as attending to the subtle movement cues of others during social interaction (Gowen, 2012; Dawson and Toth, 2015; Chevalier et al., 2016).

Motoric differences can also alter the production of the dynamic, moment-to-moment behavior that is integral to social communication (e.g., Brewer et al., 2016; Cook, 2016; Aransih and Edison, 2019; Bokadia et al., 2020). However, investigation of such behavior topography requires analysis at what has been described the microlevel of social interactions (e.g., Whyatt, 2017). At this level, movement pattern nuances are not easily detected via direct observation nor standardized assessments tools. Rather, they require sensors that can track the movement dynamics over time. This idea is exemplified in the work of Bokadia et al. (2020), which has revealed how movements at the microlevel of analysis unfold during the semi-structured social tasks that are used in the Autism Diagnostic Observation Schedule (ADOS, Lord et al., 2012). By applying wearable sensors on examiner-examinee dyads, with the ADOS as a social platform, the authors have revealed fluctuations in the strength and coherence of the dyads that would not have been captured by the standard ADOS algorithmic scoring. In addition, their analysis uncovered an interesting mirroring effect that contributed to a bias in the way examiners lead their interactions with neurotypical examinees versus how they lead interactions with autistic examinees. Finally, they demonstrated that a microlevel analysis of movement was more sensitive at detecting male versus female differences in social interaction patterns than observational scoring. Thus, a microlevel analysis of movement can unveil yet to be discovered phenotypes of autism.

Along these lines, studies have also begun using movement tracking methods to quantify a variety of kinematic differences in individuals with ASD (e.g., Crippa et al., 2015; Foster et al., 2020; Vabalas et al., 2020; Cavallo et al., 2021). Rinehart and colleagues, for example, examined the kinematics of movements in a group of children diagnosed with ASD (DSM-V criteria level 1), a group of children who were at the time diagnosed with Asperger’s Syndrome (now classified under the broader category of autism spectrum disorder in the latest diagnostic criteria), and a group of neurotypical children (Rinehart et al., 2006). They used a target aiming task involving three difficulty levels. Movements were tracked as the children moved a stylus on a tablet either toward left or right circular targets. For all levels, several metrics from the horizontal and vertical components of the movements were extracted which included metrics of movement preparation time, total movement time, and the shape of the movement trajectory indexed by an asymmetry ratio.

The ASD group and the Asperger’s Syndrome group showed more pronounced deficits in movement preparation than movement execution regardless of the difficulty level of the task. In addition, the ASD group showed movement preparation deficits at all task levels, while the Asperger’s Syndrome group paradoxically showed movement preparation deficits with the simple but not the difficult tasks. Interestingly, the motor planning deficits in the ASD group appeared to resemble Parkinsonian like movement abnormalities. And the analyses of the total movement time indicated that the ASD group lacked any modulation for task expectancy, regardless of the task being predictable or not. Finally, the asymmetry ratio indicated poorer movement planning in the ASD group relative to the Asperger’s Syndrome group as seen by more time spent in the deceleration phase for expected movements than the acceleration phase.

In addition to movement planning differences, Cook et al. (2013) has demonstrated that individuals with ASD also produce more varied, jerky movements with greater acceleration and velocity fluctuations as compared to age-matched neurotypical individuals when performing repeated horizontal sinusoidal arm movements. The magnitude of these measures was also correlated with ASD severity. Other kinematic investigations have reported that the efficiency of movements is hampered in individuals with ASD. Fears and colleagues, for example, examined variability and the number of under- or over-shooting of a target while performing whole-body, goal-directed movements in children with ASD and neurotypical children (Fears et al., 2023). The task was to bring a virtual ball displayed on a large screen to a designated virtual target area appearing on the right or left side of the starting position by moving the entire body. Fears et al. (2023) reported that the time taken for completing the task was longer in the children with ASD, and they used more divergent and non-optimal paths compared to the neurotypical control group.

Additionally, the authors reported that children with ASD made similar numbers of over- and under-shoots while their neurotypical peers made more over-shoots when the target area was near and more under-shoots while the target area was far. The authors speculate that these findings are due to the lack of accurate estimation of the limits of stability, or an inability to alter the required motor strategies for achieving postural stability in children with ASD. Similar studies report that youth with ASD (7–18 years) show different whole-body movements compared to their neurotypical peers while playing active video games (Ardalan et al., 2019), particularly higher variability and entropy in their movement kinematics. Moreover, Trujillo and colleagues have demonstrated diagnostic group differences in gesture kinematics by utilizing a Microsoft Kinect to record movements while participants performed instrumental daily actions such as cutting paper with scissors (Trujillo et al., 2021). Compared to neurotypical adults, the hand movements of autistic adults showed a slower peak velocity (maximum velocity of either hand) or were characterized by a longer duration of holding a static position prior to restarting the movement (hold time). These pauses in movement are interpreted to reflect greater segmentation in the movements of autistic individuals. The authors also observed greater variability in the movement profiles for the autistic group compared to the NT group, consistent with other reports (e.g., Gowen and Hamilton, 2013).

Expanding the potential for motor behaviors as biomarkers, Wu and colleagues have made strides in characterizing neurodevelopmental levels through movement analysis in individuals with ASD. By examining hand trajectories and speed profiles during a pointing task among participants with ASD, neurotypical individuals, children, and parents of those with ASD, they introduced an R-parameter—devised via a machine learning algorithm—to quantify motor noise. This metric not only elucidates movement disturbances in ASD but also reflects the neurodevelopmental trajectory, differentiating between stages of maturity and varying ASD severity. The R-parameter, indicative of nuanced motor control, aligns jerkier movements and slower speeds with increased ASD severity, thus reinforcing movement analysis as a nuanced lens through which the severity and developmental aspects of ASD may be understood (Wu et al., 2018).

Continuing from their machine learning innovations, Wu et al. observed that the movement transitions within the ASD group did not align with the expected maturation patterns post seven years of age, highlighting a potential biomarker for developmental delays. The R-parameter’s ability to identify ASD severity also showed a significant correlation with established psychiatric assessment scores, strengthening its validity as a diagnostic tool. Furthermore, the detection of movement disturbances in the parents of ASD participants implies a filial component, suggesting that such kinematic differences may serve as subtle indicators of the broader autism phenotype. This data-driven methodology underscores the value of movement analysis in differentiating neurodevelopmental trajectories and reinforces the concept of utilizing movement kinematics not only in identifying ASD but also in potentially screening neurodevelopmental variances within family lines.

Recent advancements in understanding movement kinematic differences in ASD underscore a compelling avenue of inquiry: the potential association between such kinematic differences and levels of autistic traits within the neurotypical population. Notably, autistic traits, which can reflect a spectrum of social, communicative, and behavioral features, can manifest in varying degrees of intensity. These traits, even in their milder, sub-clinical form, have been conceptualized as nuanced expressions of features often associated with autism (Folstein and Rutter, 1977). This spectrum, reflective of the broader autism phenotype (BAP), suggests that autistic traits are not necessarily confined to those with a clinical diagnosis of ASD but can extend into the neurotypical population, presenting as attenuated, autistic-like qualities. The exploration of these traits in relation to their kinematic expressions presents a unique opportunity to broaden our understanding of how movement can be influenced by autistic traits.

To our knowledge, there is only one study that has examined links between the BAP and movement patterns in neurotypical individuals. Granner-Shuman and colleagues recorded the hand movements of neurotypical college students during a motor coordination task with a research assistant (Granner-Shuman et al., 2021). The authors defined interactional synchrony as the degree to which the form of the hand and fingers was symmetrical between the participant and research assistant. Measures for the motor planning task included the start time of movement and the time and magnitude of the maximum speed. Autistic traits were measured using the Autism Spectrum Quotient (AQ, Baron-Cohen et al., 2001) with a particular focus on the communication subscale. Results revealed significant negative correlations between communication skills and interactional synchrony, and between communication skills and movement start time. Mediation models additionally revealed that relationships between AQ Communication and interactional synchrony were mediated by motor functioning. The authors suggest that those with higher levels of autistic traits tend to form motor plans and execute the subsequent movements faster, which they argue is linked to a reduced ability to synchronize with others.

Additional studies highlight the role of AQ subscales might play in the nuanced variations in biological motion perception and the neural underpinnings of social cognition. These domains are linked to observable behavioral distinctions and neural activity. For example, an elevation in autistic-like traits within the realm of communication has been correlated with a diminished capacity for the local processing of biological motion (Wang et al., 2022), potentially reflecting a hindered ability to leverage prior social experiences in anticipating the movements of others, as posited by Puglia and Morris (2017). Such insights underscore the role of distinct autistic traits, especially those pertaining to social skills and communication, in understanding social interaction dynamics. Furthermore, the subscales addressing attention switching and imagination have also been identified as significant contributors to neural differences during the perception of biological motion (see Puglia and Morris, 2017; Hudson et al., 2023).

In the current landscape of biological motion research in autism, a significant focus has been placed on the perceptual dimensions. However, our investigation places an emphasis on how autistic traits might manifest in the microlevel structure of biological movements. By integrating AQ subscales into our analytical framework, we seek to not only broaden our understanding but also pave the way for new hypotheses and insights into the interplay between the different types of autistic traits and nuanced micromovement expressions. The AQ is divided into subscales tapping into 5 trait constructs associated with autism: communication, social skills, attention to detail, attention switching, and imagination. Higher scores on each subscale reflect elevated levels of autistic traits for that construct. We posit that each of these traits may or may not play a role in idiosyncrasies in micro-movement patterns during social expressions. For example, questions regarding communication and social skills assess an individual’s proficiency and comfort in social settings. Elevated scores in these subscales could influence the nuances of social movement kinematics, as they reflect the individual’s ability to interpret social cues and respond with appropriate expressivity. The attention to detail subscale captures the degree to which individuals notice and prioritize subtle and specific aspects of their environment, such as intricate patterns, faint sounds, and minute details often missed by others. This heightened perceptual sensitivity could also influence the intricacy of social movement production. Individuals with elevated scores on this subscale could exhibit unique motor patterns influenced by a more nuanced perception of the world.

Attention switching taps into an individual’s flexibility in transitioning between activities and conversations, even deviating from pre-established plans. This cognitive flexibility is likely relevant to the context of social movement production in that individuals who show elevated scores in attention switching may manifest differential movement dynamics in real-time, which is crucial given the fluid nature of social exchanges. Finally, the imagination subscale focuses on one’s ability to effortlessly imagine and understand others’ experiences, whether during pretend games or while reading stories. This ability to mentally simulate and empathize with diverse social experiences possibly plays a role in shaping the production of social body movements. Individuals with elevated scores on the imagination subscale may have unique internal models of movements (Wolpert et al., 1995). Consequently, variations in this subscale could lead to a more nuanced understanding of the links between imagination and the articulation of internal states through movement. Thus, by including AQ subscales we can examine which specific BAP features that comprise the AQ (if any) play a more dominant role in modulating movement idiosyncrasies.

In this paper, we unveil our initial findings from a comprehensive study examining mappings between the BAP and whole-body kinematics during emotional expressions. These insights are part of an ambitious ongoing project aimed at harnessing visual intelligence in human movement analysis as a potential digital phenotyping approach for autism. Our research is driven by the understanding that movement, as a direct reflection of neurological processes, provides a distinctive lens through which we can explore the nuanced variations in movement expressiveness that span the endophenotypic spectrum. Building on existing research that delineates movement discrepancies in individuals with ASD and their implications for social communication, we hypothesized a linear relationship between heightened levels of broader autism phenotype (BAP) traits in neurotypical individuals and the manifestation of idiosyncratic kinematic features. However, given the exploratory nature of our study, and the scarcity of prior research directly investigating these specific relationships, we did not posit the explicit directionality of the linear relationships between BAP traits and kinematic features. Our aim was to illuminate potential patterns and associations that may lay the groundwork for future focused and hypothesis-driven inquiries.

## 2 Materials and methods

### 2.1 Biological movements repository

A repository of point-light display recordings was furnished by volunteer study participants (*N* = 58). The participants were college students within the age range of 18 to 33 years. We specifically targeted college students to obtain a sample with minimal age-related variability in movement patterns, which could potentially confound the investigation of specific kinematic features of interest. The sample age range is considered to represent a period of optimal kinematic efficiency in bodily movements among neurotypical individuals (Hines Woollacott and Shumway-Cook, 1990; Honda et al., 2022; Muschter et al., 2023). The participants were recruited via course announcements, campus online listservs, and community flyers. Each participant received an electronic gift card of small monetary value for their participation. All aspects of this study have been approved by the Human Subjects Institutional Review Board of Indiana University. Participant characteristics are summarized in Table 1.

**Table 1:** Sample characteristics.

Characteristic	*N* = 58^1^
Age	(22.0, 21.0, 3.3, 18.0, 33.0)
Sex	
Female	47 (81%)
Male	11 (19%)
Autism Spectrum Quotient (AQ)	(20.3, 20.5, 6.10, 4.00, 32.0)
AQ: Communication	(3.26, 3.50, 2.34, 0.00, 9.00)
AQ: Social skills	(3.91, 4.00, 2.45, 0.00, 9.00)
AQ: Attention to detail	(5.38, 5.00, 1.99, 1.00, 9.00)
AQ: Attention Switch	(5.36, 5.50, 1.97, 2.00, 10.0)
AQ: Imagination	(2.59, 2.00, 1.55, 0.00, 6.00)

1 Mean, Median, SD, Range; *n* (%).

The point-light displays (PLDs) in our repository are a configuration of moving points that depict human biological motion (Johansson, 1973). We chose to use PLDs in our study because we wanted to isolate movement from other pictorial cues that are present in standard video to simplify the movement analysis. The biological movements in the repository depicted different emotional expressions. Emotional expressions were selected because they contain a variety of movement kinematics to convey valence and intensity. Many emotional states are communicated through the whole body, including both vertical and horizontal movement, though arm movements and elbow flexion may be particularly informative (De Meijer, 1989; Sawada et al., 2003; Dael et al., 2013; Poyo Solanas et al., 2020). Only a subset of the emotion PLDs from the repository were included in our analyses. They were angry, happy, and fearful. In addition, a social functioning profile, linked to each participant, was compiled from a list of self-report instruments that are in line with the NIH Research Domain Criteria (RDoC) of social processes. In this paper, we focus on the BAP exclusively, which was assessed via *the Autism Spectrum Quotient* (AQ; Baron-Cohen et al., 2001); a self-administered 50-item questionnaire. The scores obtained on the AQ reflect the level of one’s autistic traits. Scores on the AQ can range from 0 to 50, where higher scores reflect more autistic traits, and 0–10 for the 5 subscales (described in the Introduction).

### 2.2 Generating point-light displays

PLDs were created using a motion capture setup consisting of a Microsoft Kinect camera and the Kinect-based biological motion capture (KBC) toolbox (Shi et al., 2018). The KBC toolbox captures depth information and body frame data at a sampling rate of 30 Hz thus recreating the participants’ biological movements in three-dimensional space. All PLDs produced with KBC included 25 points which mark crucial points such as head, shoulders, elbows, hands, spine, hips, knees, and feet. Recording each PLD involved having the participant stand in front of the Kinect camera mounted on a tripod and adjusted to about 1.4 meters. Once the Kinect captured the participant, a tracking point-light figure was displayed in the preview window of the KBC GUI. At that point, we made sure the participant’s entire body was centered within the frame by adjusting the Kinect camera’s angle and/or location to approximate an aspect ratio of 1:1 for each PLD recording. Once centered within frame, the participants were then explained that they were going to perform several discrete whole-body movements. They were instructed to try to remain in their position while acting out the movements to remain centered within the camera frame. Then an experimenter read aloud a brief prompt such as, “After returning from a weekend getaway, you find that your roommate has trashed your apartment and completely ignored your agreements, which makes you very angry.” After the prompt, the experimenter said the word “action” which signaled to the participants to perform the movements. The prompts were adapted from Scherer and Ellgring (2007). They were intended to prime the participants before producing each movement. A distinct prompt was read for each emotional movement type. No additional guidance was provided to the participants to allow for variability in their movements (e.g., Ma et al., 2006). The recording order for each PLD was randomized across subjects.

### 2.3 KBC data preprocessing

The KBC toolbox generates output in text file format. The files store spatial information for all the 25 points of the participant body represented by multiple rows containing a vector of x-y coordinates, with the x and y values denoting the spatial positions in the 2D plane. We note here that these coordinates are Cartesian coordinates for the pixel location of each tracked point, reflecting the locations of the tracked fiducial points in a two-dimensional space. The KBC tool facilitates the recording of these coordinates for subsequent analysis and interpretation in the context of biological motion research. Each subsequent row in the text file represents the next frame in the PLD recording.

Preprocessing of these output files was employed to attenuate several artifacts in the data. The first issue we encountered was several instances of recording overlap which resulted in duplicated data within a single text file. This issue was mitigated with visual inspection of the PLD to determine where the duplication occurred and then removal of any redundant data in the text file. Secondly, there were scenarios where the subject moved too close to the Kinect sensor during recording. This resulted in overflow-induced anomalies that manifested as large negative coordinate values. Instances of overflow-induced anomalies, although rare, were identified via visual inspection and the coordinate data from missing fiducial points within frames displaying overflow were removed. We also observed incomplete and/or missing PLD points in the text files resulting from the KBC failing to capture specific points during recording sessions. In all cases, we applied a region-of-interest (ROI) method to reduce uncertainty in the location of body regions by including multiple tracked points within each ROI. Where only one point was missing from the ROI, the center of mass (CoM) of the ROI was calculated with the remaining points. Where more than one point within the ROI was missing, the ROI was marked as missing and removed from subsequent analysis. Finally, spurious signals, often arising from the initial and final movements captured by the camera, introduced noise to the dataset. To effectively mitigate these artifacts, a trapezoidal windowing function-based filtering technique was implemented. This procedure involved multiplying the extracted kinematic time-series by a trapezoidal signal to attenuate changes in the first (and last) 10% of data in every observation.

### 2.4 Defining regions of interest in the PLDs

**Figure 1:**
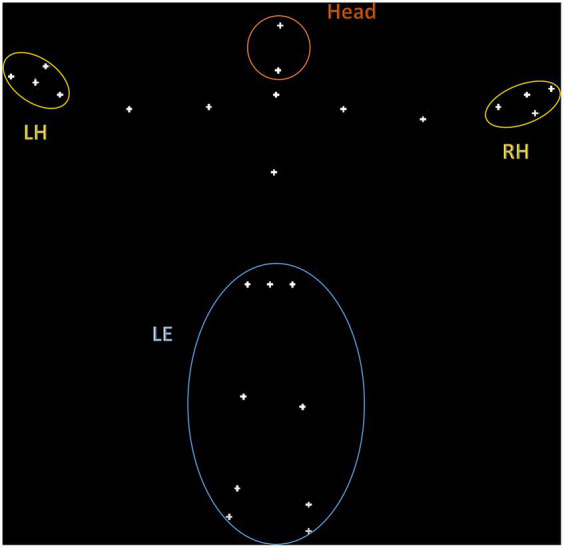
Visualization of the 4 regions of interest (ROIs) defined based on the fiducial points labeled in the KBC toolbox: lower extremities (LE), right and left hand (RH and LH), and the head.

As shown in Figure 1, a total of 4 regions of interest (ROIs) were defined based on the fiducial points labeled in the KBC toolbox: lower extremities (LE), right and left hand (RH and LH), and the head. As described in the results, our linear mixed-effects models included nested observations across these ROIs, with the ‘LE’ ROI used as the reference level in the model output. The ‘LE’ encompasses points corresponding to the legs and hips, and thus the CoM of this region reflects large scale motion of the subject in space. The ‘LE’ region shows markedly lower magnitude of displacement compared to, for example, the hand regions. Thus, we refer our smaller ROI kinematics to this larger, more stable ROI, to ground the results in the most global measure of movement. Prior movement studies have focused on similar ROIs, such as gait (Gong et al., 2020), balance (Kohen-Raz et al., 1992; Memari et al., 2014; Bojanek et al., 2020), upper limb movements (Mari et al., 2003; Wedyan and Al-Jumaily, 2016; Gamez Corral et al., 2020), and head movements (Vabalas et al., 2020). Differences in the postural control and upper limb movements have differentiated between ASD and neurotypical individuals (Lim et al., 2017; Fears et al., 2023).

### 2.5 Digitized kinematics

**Figure 2:**
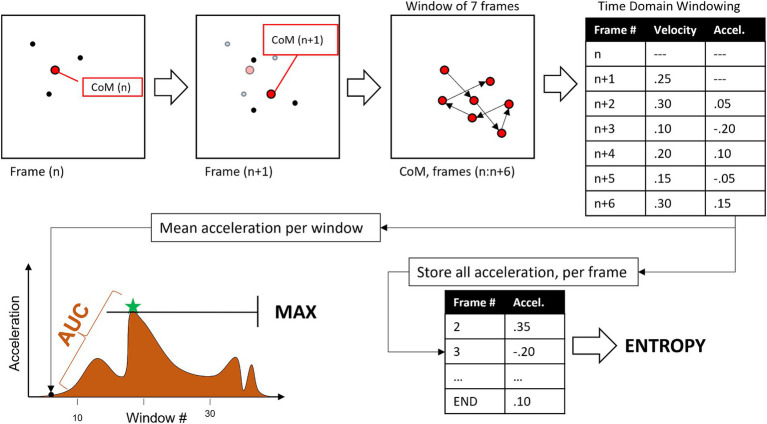
Flow diagram for extraction of kinematic features from each point light display (PLD).

Three digital kinematic features were extracted from the acceleration quantified in the PLDs: (1) entropy, (2) area under the curve (AUC), and (3) maximum acceleration (MAX). These measures were computed through the following sequence, illustrated in Figure 2:

1. KBC coordinates were first normalized by dividing them by the distance between “Hip” edge points in the first 3 frames, to adjust for size of body in the frame of the camera.
2. Within each frame, the coordinates of the included points were used to calculate the CoM for each of seven ROIs.
3. Velocity (magnitude of CoM change between frames) and then acceleration (change in velocity) were quantified from discrete sets of 7 frames.
4. Mean Abs (Acceleration) across the 7 frames is stored for use in quantifying MAX and AUC.
  - (not in figure) acceleration values for the initial and final 10% of frames are multiplied by a triangle ramp to keep them close to 0 to account for instability in initial estimate of fiducial points by KBC.
5. All acceleration values are stored for quantifying entropy.
6. Digitized kinematics (per ROI, per emotional movement).
  - entropy = sample entropy for all accelerations {tolerance = 0.2 * SD, embedding dimension = 3}
  - AUC = integral of the windowed acceleration time-series.
  - MAX = largest windowed acceleration.

The Center of Mass (CoM) was computed as a weighted average of the spatial coordinates of all tracked points in each region of interest (ROI). In our analysis, all points were assigned equal weight, with each point given a mass value of 1. The calculation involved summing the products of the individual point coordinates (x, y) and their corresponding mass values, and then dividing them by the total mass. The formula for the CoM calculation is as follows:


$$CoM_{x}=\frac{\sum_{i=1}^{n}\left(x_{i}\times mass_{i}\right)}{\sum_{i=1}^{n}mass_{i}}$$

$$CoM_{y}=\frac{\sum_{i=1}^{n}\left(y_{i}\times mass_{i}\right)}{\sum_{i=1}^{n}mass_{i}}$$

where n is the number of points in the ROI, $x_{i}$ , $y_{i}i\;$ are the coordinates of the i -th point, and all points are considered to have equal weight.

Acceleration, as the derivative of velocity, captures alterations in motion speed. It highlights sudden changes in movement, shedding light on the nuanced dynamics of emotional expression. Entropy quantifies the regularity or irregularity of acceleration magnitudes across the emotion expression. It provides insights into the complexity and unpredictability of motion patterns. A low entropy value indicates that the acceleration pattern exhibits a high degree of regularity and consistency. A high entropy value suggests that the accelerations are highly variable and unpredictable within the expression, signifying a more complex and irregular motion pattern. The AUC and MAX aspects of acceleration capture oblique information on the distribution of motion energy across the expression.

By quantifying these kinematic features from the displacement vectors, we gain a multidimensional understanding of how emotions manifest through body movement. This approach transcends the boundaries of visual perception, deepening our comprehension of the human emotional experience encoded in motion. These kinematic features, when considered collectively, provide a quantitative foundation for comprehensive analysis and comparison across various emotional states.

## 3 Results

To explore whether BAP features can predict the expression of idiosyncratic micro-movements, we first investigated the simple linear dependence of each digitized kinematic on each BAP feature separately for each ROI and TASK. Multiple significant correlations were observed for each of the three digitized kinematics. Several of the effects were observed across ROIs. These significant associations are for measures that are ultimately nested within subject both across the ROIs and the TASKs. Therefore, these linear regressions are not appropriate for describing the nuanced relationship of BAP to movement across the body. Nevertheless, the number and strength of these relationships suggested that a nuanced model could capture the overall pattern of BAP features to movement parameters.

To fully describe the results, linear mixed-effects models (LMEM) were employed that could account for the nested observations and repeated measures of movement. We fitted a series of LMEM (estimated using REML and nloptwarp optimizer) to examine the relationship between different digitized kinematics derived from the PLDs and different BAP features (i.e., autistic traits). We operationally defined BAP features from the scores obtained from the AQ (including subscale scores). Thus, 6 different BAP features and 3 target kinematic measures were examined. Table 2 lists the BAP features and target kinematic measures.

**Table 2:** List of the predictive Broad Autism Phenotype (BAP) features and the targets of the models.

BAP features (predictors)	kinematic measures (targets)
AQ total	Entropy
Communication	Area under curve (AUC)
Social skills	Maximum acceleration (MAX)
Attention to detail	
Attention switch	
Imagination	

Each feature was fitted to each of the three targets in separate models. This resulted in 21 different models.

Each LMEM had only one BAP predictor to maintain simplicity of the model and since the BAP features are collinear. Moreover, each BAP feature was used in three different LMEM—predicting different target kinematic measures (entropy, AUC, and MAX). The result was a total of 18 models. Included in the models were interactions terms between the BAP feature and the 3 emotion tasks, and the BAP feature and the 4 ROIs. In addition, the models included an age term, aiming to account for any residual age-related variations within our sample, and a sex term to control the influence of sex on our findings. The model structure was random intercepts for each participant and random slopes for emotion task within each participant. The use of 3 emotion PLDs containing 4 ROIs generated 12 observations per participant, resulting in 696 discrete observations. However, the sample contained observations with missing values due to recording equipment errors; therefore, the sample was further reduced to 676 observations. The function for each LMEM can be describe as follows:

$\begin{array}{l} DK_{ijk}=b_{0}+b_{1}BAP_{ijk}+b_{2}\;Sexb_{ij}+b_{3}\;Age_{ij}+b_{4}BAP_{ijk}\;x\;Task_{ijk}\\ \;\;\;\;\;\;\;\;\;\;\;\;\;\;\;+b_{5}BAP_{ijk}\;x\;ROI_{ijk}+u_{0j}+u_{1j}\;x\;Task_{ijk}+e_{ij} \end{array}$

Where:

- *DK_ij_*: Digitized kinematic measure *k* for observation *i* from participant *j*
- *BAP_ijk_*: BAP feature *k* for observation *i* from participant *j*
- *Sex_ij._*: Sex term for observation *i* from participant *j*
- Age*_ij_*: Age term for observation *i* from participant *j*
- *Task_ij_* - > {fear, anger, happy}: Emotion expression task *k* for observation *i* from participant *j*
- *ROI_ij_* - > {lower extremities, head, left hand, right hand}: Region of interests in the PLD for observation *i* from participant *j*
- *b_0_*, *b*_*1*,_ …, *b_3_*: Fixed effects coefficients
- *u_0j_*: Random intercept for *Task* with participant *j*
- *u_1j_*: Random slope for participant *j*
- *e_ij_*: Residual for observation *i* from participant *j*

We used R (R Core Team, 2023) and *lme4* (Bates et al., 2014) to perform the LMEM analyses. Since we were only interested in exploring the predictability of kinematics from features of the BAP, we focus here only on the results in which a BAP features and interactions with BAP features significantly predicted a kinematic measure at a p-level less than 0.05. In addition, we performed a Holm-Bonferroni correction (Holm, 1979) for all *p*-values using the *p.adjust* function in *R*. This correction method adjusts the significance thresholds for all the multiple comparisons. A visual inspection of residual plots revealed deviations from homoscedasticity and normality for the AUC and MAX measures. These measures were therefore log-transformed prior to model fitting. However, there were no obvious deviations from homoscedasticity or normality for the entropy measures. Likelihood ratio tests comparing each of the 18 models indicated significantly better fitting models compared to a null model without any BAP feature as a predictor. However, BAP feature interactions with ROI also significantly improve the model fit. Therefore, the subsequent sections focus on the results of the beta coefficients, with a specific consideration of the fixed effects of each BAP feature and its interactions with Task and ROI to potentially predict digitized kinematics derived from human biological motion stimuli.

### 3.1 Autism Spectrum Quotient (AQ) and kinematic features

With respect to the relationship between Autism Spectrum Quotient (AQ) total scores and kinematic features, Holm-Bonferroni adjusted *p* values revealed that the relationship between AQ scores and Entropy was not significant at the reference level (β = 0.01, *p* = > 0.9). Conversely, at the reference level, for AUC and MAX, the negative coefficients (β = −0.05 for both, *p* = 0.021 for AUC and *p* = 0.005 for MAX) indicate that as autistic traits increase, the overall volume and peak of acceleration in their movements decreases. Negative coefficients, relative to the reference level, for AQ interactions with three ROIs in the context of Entropy (Head: β = −0.01, LH: β = −0.02, RH: β = −0.03; all *p* < 0.001) showed that higher AQ scores were associated with decreased ordered movements, particularly in these body regions. In contrast, positive coefficients for AUC and MAX (ranging from β = 0.02 to 0.10; all *p* < 0.001) highlight that autistic traits correlate with increased movement in these same regions. Across all models, interaction effects of AQ score with Task were not significant. There were also no significant effects based on sex and age. Results are presented in Table 3.

**Table 3:** Mixed effects model results for total score of the Autism Spectrum Quotient (AQ) as the feature.

	Entropy			AUC			MAX		
Feature	Beta	95% CI^a^	*p*-value (adjusted)^b^	Beta	95% CI^a^	*p*-value (adjusted)^b^	Beta	95% CI^a^	*p*-value (adjusted)^b^
AQ	0.01	0.00, 0.02	0.013 (>0.9)	−0.05	−0.08, −0.02	0.002 (0.021)	−0.05	−0.08, −0.03	<0.001 (0.005)
Sex									
Female	—	—		—	—		—	—	
Male	−0.12	−0.27, 0.03	0.10 (>0.9)	0.23	−0.27, 0.73	0.4 (>0.9)	0.17	−0.19, 0.53	0.3 (>0.9)
Age	0.00	−0.02, 0.01	0.7 (>0.9)	0.05	−0.01, 0.11	0.13 (>0.9)	0.02	−0.02, 0.07	0.3 (>0.9)
AQ * ROI									
AQ * head	−0.01	−0.02, −0.01	<0.001 (<0.001)	0.02	0.02, 0.03	<0.001 (<0.001)	0.03	0.02, 0.03	<0.001 (<0.001)
AQ * LH	−0.02	−0.03, −0.02	<0.001 (<0.001)	0.10	0.09, 0.11	<0.001 (<0.001)	0.10	0.10, 0.11	<0.001 (<0.001)
AQ * RH	−0.03	−0.03, −0.02	<0.001 (<0.001)	0.10	0.09, 0.11	<0.001 (<0.001)	0.10	0.10, 0.11	<0.001 (<0.001)
AQ * task									
AQ * anger	0.00	−0.01, 0.00	0.4 (>0.9)	0.01	0.00, 0.02	0.2 (>0.9)	0.00	−0.01, 0.01	0.9 (>0.9)
AQ * happy	0.00	0.00, 0.01	0.6 (>0.9)	0.00	−0.01, 0.01	0.9 (>0.9)	0.00	−0.01, 0.01	0.7 (>0.9)

a CI = Confidence interval.

b Holm.

AUC = area under curve, MAX = maximum acceleration (MAX), ROI = region of interest, LH = left hand, RH = right hand. Reference level, ROI = lower extremities (LE) and Task = fear, is the row with AQ as feature. Reference factor levels not labeled in the table.

### 3.2 Communication and kinematic features

Similarly, there were significant relationships between the Communication subscale scores, and various kinematic features represented by Entropy, AUC, and MAX metrics at the reference levels: (ROI = Lower Extremity) and (TASK = Fear). Notably, a positive beta coefficient (0.07) with a significant *p*-value (0.005) in the context of Entropy suggests that the more neurodivergent the participant’s communication style was, greater randomness or variability manifested in their movements. Conversely, negative beta coefficients for AUC (−0.18) and MAX (−0.20), significant at *p* = 0.014 and *p* < 0.001, respectively, indicate that elevated scores on the communication subscale correlate with lower peak values and less area under the curve in movements, potentially reflecting more restrained motion. Further analysis reveals significant interactions between communication scores and specific ROIs. For instance, elevated communication scores predicted a decrease in Entropy, relative to reference level, in all three ROIs (Head: β = −0.06, LH: β = −0.11, RH: β = −0.12, all *p* < 0.001). The positive beta values for AUC and MAX across these ROIs, relative to the reference, indicate that the volume and intensity of movements in these ROIs tend to increase with elevated communication subscale scores. No significant interaction effects of communication skills with Task were observed. There were also no significant effects based on sex and age. Results are presented in Table 4.

**Table 4:** Mixed effects model results for the communication subscale of the Autism Spectrum Quotient (AQ) as the feature.

	Entropy			AUC			MAX		
Feature	Beta	95% CI^a^	*p*-value (adjusted)^b^	Beta	95% CI^a^	*p*-value (adjusted)^b^	Beta	95% CI^a^	*p*-value (adjusted)^b^
Communication	0.07	0.04, 0.10	<0.001 (0.005)	−0.18	−0.27, −0.09	<0.001 (0.014)	−0.20	−0.27, −0.13	<0.001 (<0.001)
Sex									
Female	—	—		—	—		—	—	
Male	−0.12	−0.27, 0.02	0.10 (>0.9)	0.27	−0.21, 0.76	0.3 (>0.9)	0.21	−0.15, 0.56	0.2 (>0.9)
Age	0.00	−0.02, 0.01	0.7 (>0.9)	0.05	−0.01, 0.11	0.084 (>0.9)	0.03	−0.02, 0.07	0.2 (>0.9)
Communication * ROI									
Communication * head	−0.06	−0.08, −0.04	<0.001 (<0.001)	0.11	0.07, 0.16	<0.001 (<0.001)	0.12	0.07, 0.17	<0.001 (<0.001)
Communication * LH	−0.11	−0.13, −0.09	<0.001 (<0.001)	0.45	0.40, 0.50	<0.001 (<0.001)	0.46	0.41, 0.51	<0.001 (<0.001)
Communication * RH	−0.12	−0.14, −0.10	<0.001 (<0.001)	0.44	0.39, 0.49	<0.001 (<0.001)	0.45	0.40, 0.50	<0.001 (<0.001)
Communication * task									
Communication * anger	−0.01	−0.03, 0.01	0.5 (>0.9)	0.04	−0.01, 0.10	0.12 (>0.9)	0.02	−0.03, 0.06	0.5 (>0.9)
Communication * happy	0.00	−0.02, 0.02	>0.9 (>0.9)	−0.01	−0.06, 0.05	0.8 (>0.9)	−0.02	−0.06, 0.03	0.4 (>0.9)

a CI = Confidence interval.

b Holm.

AUC = area under curve, MAX = maximum acceleration (MAX), ROI = region of interest, LH = left hand, RH = right hand. Reference level, ROI = lower extremities (LE) and Task = fear, is the row with AQ as feature. Reference factor levels not labeled in the table.

### 3.3 Social skills and kinematic features

At reference level, positive beta coefficient (β = 0.06, *p* = 0.003) for the relationship between social skills and entropy indicates that individuals with higher scores in the social skills domain of the AQ tend to have greater complexity in their emotional movement patterns. Conversely, significant negative coefficients for both AUC (β = −0.23, *p* < 0.001) and MAX (β = −0.22, *p* < 0.001) reflect that elevated social skills scores are associated with decreased overall volume and peak movement values, respectively. Examination of interactions between social skills and specific ROIs revealed consistent patterns. Negative coefficients, relative to reference level, for entropy across all ROIs (Head: β = −0.05, LH: β = −0.09, RH: β = −0.10; all *p* < 0.001) suggest that higher social skills subscale score correlate with less randomness or more structured movements in these regions. Positive coefficients for AUC and MAX in these ROIs indicate that individuals with elevated autistic features in the social skills domain, reflective of a more neurodivergent social style, may engage in more pronounced movements. For all models, there were no significant sex differences or age effects. Furthermore, the interaction effects of social skills with specific tasks (anger and happiness) were not statistically significant. Results are presented in Table 5.

**Table 5:** Mixed effects model results for the social skills subscale of the Autism Spectrum Quotient (AQ) as the feature.

	Entropy			AUC			MAX		
Feature	Beta	95% CI^a^	*p*-value (adjusted)^b^	Beta	95% CI^a^	*p*-value (adjusted)^b^	Beta	95% CI^a^	*p*-value (adjusted)^b^
Social skills	0.06	0.03, 0.08	<0.001 (0.003)	−0.23	−0.32, −0.14	<0.001 (<0.001)	−0.22	−0.29, −0.16	<0.001 (<0.001)
Sex									
Female	—	—		—	—		—	—	
Male	−0.12	−0.26, 0.03	0.12 (>0.9)	0.22	−0.28, 0.72	0.4 (>0.9)	0.16	−0.20, 0.52	0.4 (>0.9)
Age	0.00	−0.02, 0.01	0.7 (>0.9)	0.05	−0.01, 0.11	0.13 (>0.9)	0.02	−0.02, 0.07	0.3 (>0.9)
Social skills * ROI									
Social skills * head	−0.05	−0.07, −0.03	<0.001 (<0.001)	0.10	0.06, 0.14	<0.001 (<0.001)	0.11	0.06, 0.15	<0.001 (<0.001)
Social skills * LH	−0.09	−0.11, −0.07	<0.001 (<0.001)	0.40	0.36, 0.44	<0.001 (<0.001)	0.41	0.37, 0.45	<0.001 (<0.001)
Social skills * RH	−0.10	−0.12, −0.08	<0.001 (<0.001)	0.39	0.35, 0.43	<0.001 (<0.001)	0.41	0.37, 0.45	<0.001 (<0.001)
Social skills * task									
Social skills * anger	−0.01	−0.03, 0.00	0.12 (>0.9)	0.04	0.00, 0.09	0.074 (0.96)	0.02	−0.02, 0.05	0.4 (>0.9)
Social skills * happy	0.00	−0.02, 0.02	>0.9 (>0.9)	0.00	−0.05, 0.05	>0.9 (>0.9)	−0.01	−0.05, 0.03	0.6 (>0.9)

a CI = Confidence interval.

b Holm.

AUC = area under curve, MAX = maximum acceleration (MAX), ROI = region of interest, LH = left hand, RH = right hand. Reference level, ROI = lower extremities (LE) and Task = fear, is the row with AQ as feature. Reference factor levels not labeled in the table.

### 3.4 Attention to detail and kinematic features

The positive coefficient for entropy (β = 0.06, *p* = 0.018) at the reference level suggests that higher subscale scores in attention to detail are associated with increased complexity or randomness in movement patterns. Scores on the attention to detail subscale were not associated with AUC at reference. The significant negative coefficients for MAX (β = −0.20, *p* < 0.001) indicate that elevated attention to detail sores correspond with lower overall peak values. This could reflect a more controlled or restrained motor output among individuals who focus intensively on details. Negative coefficients for entropy in these specific ROIs (Head: β = −0.05, LH: β = −0.09, RH: β = −0.10; all *p* < 0.001) reveal that higher attention to detail is linked with less variability in these specific areas. The positive coefficients for AUC and MAX (ranging from β = 0.09 to 0.37; all *p* < 0.001) further suggest that individuals with high attention to detail may engage in more pronounced movements. For all models, no significant sex differences or age effects were found. Interaction effects between attention to detail scores and Task was not significant. Results are presented in Table 6.

**Table 6:** Mixed effects model results for the attention to detail subscale of the Autism Spectrum Quotient (AQ) as the feature.

	Entropy			AUC			MAX		
Characteristic	Beta	95% CI^a^	*p*-value (adjusted)^b^	Beta	95% CI^a^	*p*-value (adjusted)^b^	Beta	95% CI^a^	*p*-value (adjusted)^b^
Attention to detail	0.06	0.03, 0.10	<0.001 (0.018)	−0.17	−0.27, −0.07	0.001 (0.1)	−0.20	−0.27, −0.12	<0.001 (<0.001)
Sex									
Female	—	—		—	—		—	—	
Male	−0.12	−0.27, 0.03	0.12 (>0.9)	0.26	−0.25, 0.76	0.3 (>0.9)	0.18	−0.19, 0.54	0.3 (>0.9)
Age	0.00	−0.02, 0.01	0.7 (>0.9)	0.05	−0.01, 0.11	0.12 (>0.9)	0.02	−0.02, 0.07	0.3 (>0.9)
Attention to detail * ROI									
Attention to detail * head	−0.05	−0.06, −0.04	<0.001 (<0.001)	0.09	0.06, 0.11	<0.001 (<0.001)	0.10	0.07, 0.12	<0.001 (<0.001)
Attention to detail * LH	−0.09	−0.10, −0.08	<0.001 (<0.001)	0.36	0.34, 0.39	<0.001 (<0.001)	0.37	0.35, 0.40	<0.001 (<0.001)
Attention to detail * RH	−0.10	−0.11, −0.09	<0.001 (<0.001)	0.36	0.33, 0.38	<0.001 (<0.001)	0.37	0.34, 0.40	<0.001 (<0.001)
Attention to detail * task									
Attention to detail * anger	0.00	−0.02, 0.01	0.7 (>0.9)	0.02	−0.02, 0.06	0.3 (>0.9)	0.00	−0.03, 0.04	0.8 (>0.9)
Attention to detail * happy	0.00	−0.01, 0.02	0.7 (>0.9)	0.00	−0.04, 0.04	0.9 (>0.9)	0.00	−0.03, 0.03	0.9 (>0.9)

a CI = Confidence Interval.

b Holm.

AUC = area under curve, MAX = maximum acceleration (MAX), ROI = region of interest, LH = left hand, RH = right hand. Reference level, ROI = lower extremities (LE) and Task = fear, is the row with AQ as feature. Reference factor levels not labeled in the table.

### 3.5 Attention switching and kinematic features

Although scores on this subscale did not predict entropy at the reference level, significant negative coefficients for AUC (β = −0.23, *p* < 0.001) and MAX (β = −0.20, *p* < 0.001) were observed at the reference levels showing that higher attention-switching subscale scores were associated with a reduction in acceleration volume and peak values in movements. Similarly, the interactions between attention switching scores and specific ROIs revealed negative coefficients for Entropy across these ROIs (Head: β = −0.05, LH: β = −0.09, RH: β = −0.10; all *p* < 0.001). In contrast, there were positive coefficients for AUC and MAX in these ROIs (ranging from β = 0.09 to 0.38; all *p* < 0.001), indicating attention-switching scores may drive more pronounced movements in upper ROIs. No significant sex differences or age effects emerged from the analysis. Additionally, the interaction effects of attention switching with Task did not reach statistical significance. Results are presented in Table 7.

**Table 7:** Mixed effects model results for models with the attention switching subscale of the Autism Spectrum Quotient (AQ) as the feature.

	Entropy			AUC			MAX		
Characteristic	Beta	95% CI^a^	*p*-value (adjusted)^b^	Beta	95% CI^a^	*p*-value (adjusted)^b^	Beta	95% CI^a^	*p*-value (adjusted)^b^
Attention switch	0.05	0.02, 0.08	0.002 (0.21)	−0.23	−0.33, −0.12	<0.001 (0.004)	−0.20	−0.28, −0.13	<0.001 (<0.001)
Sex									
Female	—	—		—	—		—	—	
Male	−0.12	−0.26, 0.03	0.12 (>0.9)	0.24	−0.26, 0.74	0.3 (>0.9)	0.17	−0.19, 0.53	0.4 (>0.9)
Age	0.00	−0.02, 0.01	0.7 (>0.9)	0.04	−0.01, 0.10	0.14 (>0.9)	0.02	−0.02, 0.07	0.3 (>0.9)
Attention switch * ROI									
Attention switch * head	−0.05	−0.06, −0.04	<0.001 (<0.001)	0.09	0.07, 0.12	<0.001 (<0.001)	0.10	0.07, 0.13	<0.001 (<0.001)
Attention switch * LH	−0.09	−0.10, −0.07	<0.001 (<0.001)	0.37	0.34, 0.39	<0.001 (<0.001)	0.38	0.35, 0.41	<0.001 (<0.001)
Attention switch * RH	−0.10	−0.11, −0.08	<0.001 (<0.001)	0.36	0.34, 0.39	<0.001 (<0.001)	0.38	0.35, 0.41	<0.001 (<0.001)
Attention switch * task									
Attention switch * anger	0.00	−0.02, 0.01	0.7 (>0.9)	0.01	−0.03, 0.05	0.6 (>0.9)	−0.01	−0.04, 0.02	0.6 (>0.9)
Attention switch * happy	0.01	−0.01, 0.02	0.4 (>0.9)	0.00	−0.04, 0.03	0.8 (>0.9)	−0.01	−0.04, 0.03	0.7 (>0.9)

a CI = Confidence interval.

b Holm.

AUC = area under curve, MAX = maximum acceleration (MAX), ROI = region of interest, LF = left hand, RH = right hand, LF = left foot, and RF = right foot.

### 3.6 Imagination and kinematic features

Finally, a notable positive coefficient for Entropy (β = 0.10, *p* = 0.003) at the reference level suggests that individuals with higher scores in the imagination subscale of the AQ exhibited more disorganized movement patterns (more entropy). Conversely, the substantial negative coefficients for AUC (β = −0.48, *p* < 0.001) and MAX (β = −0.44, *p* < 0.001) indicate a pronounced decrease in the overall volume and peak values of movement for individuals with elevated Imagination subscale scores. This suggests that while movements are more disorganized and varied, they are characterized by lower intensity and smaller overall motion extents. The interactions between imaginative capabilities and specific regions of interest (ROI) indicate significant negative coefficients for Entropy across these ROIs (Head: β = −0.09, LH: β = −0.15, RH: β = −0.17; all *p* < 0.001). In contrast, there were positive coefficients for AUC and MAX (ranging from β = 0.16 to 0.69; all *p* < 0.001). The modeling did not reveal significant effects based on sex and age, nor interactions between imagination scores and Tasks. Results are presented in Table 8.

**Table 8:** Mixed effects model results for the imagination subscale of the Autism Spectrum Quotient (AQ) as the feature.

	Entropy			AUC			MAX		
Feature	Beta	95% CI^a^	*p*-value (adjusted)^b^	Beta	95% CI^a^	*p*-value (adjusted)^b^	Beta	95% CI^a^	*p*-value (adjusted)^b^
Imagination	0.10	0.05, 0.14	<0.001 (0.003)	−0.48	−0.61, −0.34	<0.001 (<0.001)	−0.44	−0.54, −0.33	<0.001 (<0.001)
Sex									
Female	—	—		—	—		—	—	
Male	−0.12	−0.27, 0.03	0.11 (>0.9)	0.22	−0.27, 0.71	0.4 (>0.9)	0.17	−0.19, 0.52	0.4 (>0.9)
Age	0.00	−0.02, 0.01	0.7 (>0.9)	0.04	−0.01, 0.10	0.13 (>0.9)	0.02	−0.02, 0.07	0.3 (>0.9)
Imagination * ROI									
Imagination * Head	−0.09	−0.11, −0.06	<0.001 (<0.001)	0.16	0.10, 0.21	<0.001 (<0.001)	0.17	0.11, 0.23	<0.001 (<0.001)
Imagination * LH	−0.15	−0.18, −0.13	<0.001 (<0.001)	0.66	0.60, 0.71	<0.001 (<0.001)	0.68	0.62, 0.74	<0.001 (<0.001)
Imagination * RH	−0.17	−0.19, −0.14	<0.001 (<0.001)	0.66	0.60, 0.71	<0.001 (<0.001)	0.69	0.63, 0.75	<0.001 (<0.001)
Imagination * Task									
Imagination * anger	−0.01	−0.04, 0.02	0.6 (>0.9)	0.03	−0.04, 0.11	0.4 (>0.9)	0.00	−0.06, 0.06	>0.9 (>0.9)
Imagination * happy	0.01	−0.02, 0.04	0.5 (>0.9)	−0.01	−0.08, 0.07	0.9 (>0.9)	−0.01	−0.07, 0.05	0.7 (>0.9)

a CI = Confidence interval.

b Holm.

AUC = area under curve, MAX = maximum acceleration (MAX), ROI = region of interest, LH = left hand, RH = right hand. Reference level, ROI = lower extremities (LE) and Task = fear, is the row with AQ as feature. Reference factor levels not labeled in the table.

## 4 Discussion

In this study, we explored the nuanced interplay between autistic traits and their influence on kinematic features across neurotypical individuals. The essence of our research lay in capturing the natural, spontaneous micro-movements that individuals exhibited when asked to represent emotions, rather than the accuracy or theatrical quality of the emotional portrayal. The subtleties in the kinematics were the primary variables of interest, which did not require trained performative skills. Leveraging mixed effects modeling, our analysis unveiled significant associations between an individual’s score on the Autism Spectrum Quotient (AQ) and the order, volume, and magnitude of their emotional movement patterns. These findings expand our understanding of the intricate relationship between autistic traits and their kinematic characteristics, underscoring the broader autism phenotype’s heterogeneity across multiple levels of analysis. Notably, our results demonstrate that the various BAP constructs, tapped into by the AQ subscales, including communication, social skills, attention to detail, attention switching, and imagination, can influence the movement kinematics of emotional behaviors. Furthermore, the implications of this research extend to improving the capture and feature extraction of the movement signatures that accompany autistic traits, with the potential to refine strategies for autism phenotyping and filial screening.

Our investigation revealed several notable trends that shed light on the complex dynamics between autistic traits and movement patterns. Firstly, a consistent pattern across most autistic traits revealed that as the scores increased, movements in the lower extremities became less structured, exhibiting higher entropy. In contrast, the upper ROIs, specifically the head and hands, demonstrated more structured and predictable movements, indicating lower entropy with higher cores. Secondly, a converse relationship was observed for the AUC (indicating the volume of movement) and MAX (the peak magnitude of movement). Elevated autistic trait scores were associated with a reduction in both the volume and magnitude of movement in the lower extremities. However, in the upper ROIs, these scores tended to correlate with an increase in the volume and magnitude of movement, suggesting a more expansive range of movements in these areas for individuals with higher levels of autistic-like traits.

Thirdly, the consistency of these patterns regardless of the emotional expression being conveyed suggests that the kinematic characteristics associated with autistic traits were stable across the emotion types. This stability implies that the motor signatures identified are features of the individuals’ movement repertoire, rather than being contextually driven by the nature of the emotional task. Fourth, the variables of sex and age did not significantly contribute to the variance in our data, indicating that the kinematic patterns we observed can be attributed more confidently to the broader autism phenotype (BAP) features rather than to these demographic factors. Lastly, not all AQ subscales showed significant effects on kinematic differences. Specifically, the attention to detail and attention switching showed no effects in the LE (lower extremities) ROI regarding AUC and entropy, respectively, possibly suggesting that these autistic features have greater influence on upper body kinematics. The patterns observed in our study suggest that the kinematic idiosyncrasies associated with certain autistic traits could reflect remnants of movement-related characteristics of the broader autism phenotype (BAP), akin to traditional descriptions of social and behavioral traits initially encapsulated in the conceptualization of the BAP.

The heightened entropy, and attenuated amount and magnitude, in lower extremity movements with increasing AQ scores could reflect a greater variance in motor control or a difference in sensory-motor integration, as typically seen in autism (Fournier et al., 2010; Hannant et al., 2016; Parma and De Marchena, 2016; Amoruso et al., 2018). A significant body of literature has established that individuals with ASD often exhibit notable differences in stability and gait patterns compared to neurotypical peers (Fournier et al., 2010; Calhoun et al., 2011; Memari et al., 2014; Fears et al., 2023). These variations are characterized by increased postural sway and distinct gait patterns (Kindregan et al., 2015; Gong et al., 2020; Lum et al., 2021), pointing to a fundamental divergence in motor control and proprioceptive integration (Palmer et al., 2015; Arthur et al., 2019). Gouleme et al. (2017), for example, compared postural sway for children with ASD versus neurotypical children while they were exploring emotional faces in a lab setting. The authors reported that those with ASD showed more postural sway while exploring faces with negative emotions, particularly fear. Work by Naito et al. used accelerometer data to demonstrate that children with ASD display more movements during periods of the day when one’s body should be more still such as at night (Naito et al., 2019). The authors also showed that more movement during periods of stillness correlated with increased social impairment. Our study’s findings, which highlight increased entropy and lower AUC and MAX in the lower extremities, dovetail with these documented motor differences.

Interestingly, these associations in consideration with certain subscales could cast a light on different facets of the observed movement idiosyncrasies. For example, given that social communication involves behavior, it is not all that surprising to see the communication and social skills subscales contribute to movement idiosyncrasies. However, the observation that individuals scoring higher in the attention to detail subscale manifest a more variable and complex pattern of movements in the lower extremities (higher entropy) could possibly indicate a tendency to over-emphasize movement production during communicative behavior. Of course, this remains to be tested. Similarly, the elevated attention switching subscale scores associated with less predictable movement in the lower extremities, could possibly reflect an increased variability in moment-to-moment changes when producing social movements. Finally, elevated scores in the *Imagination* subscale were also associated with increased entropy in lower extremity movements. Such a link points to a possibility of diverging internal models (Wolpert et al., 1995) of emotional movements that could manifest through idiosyncrasies in movement kinematics.

**Figure 3:**
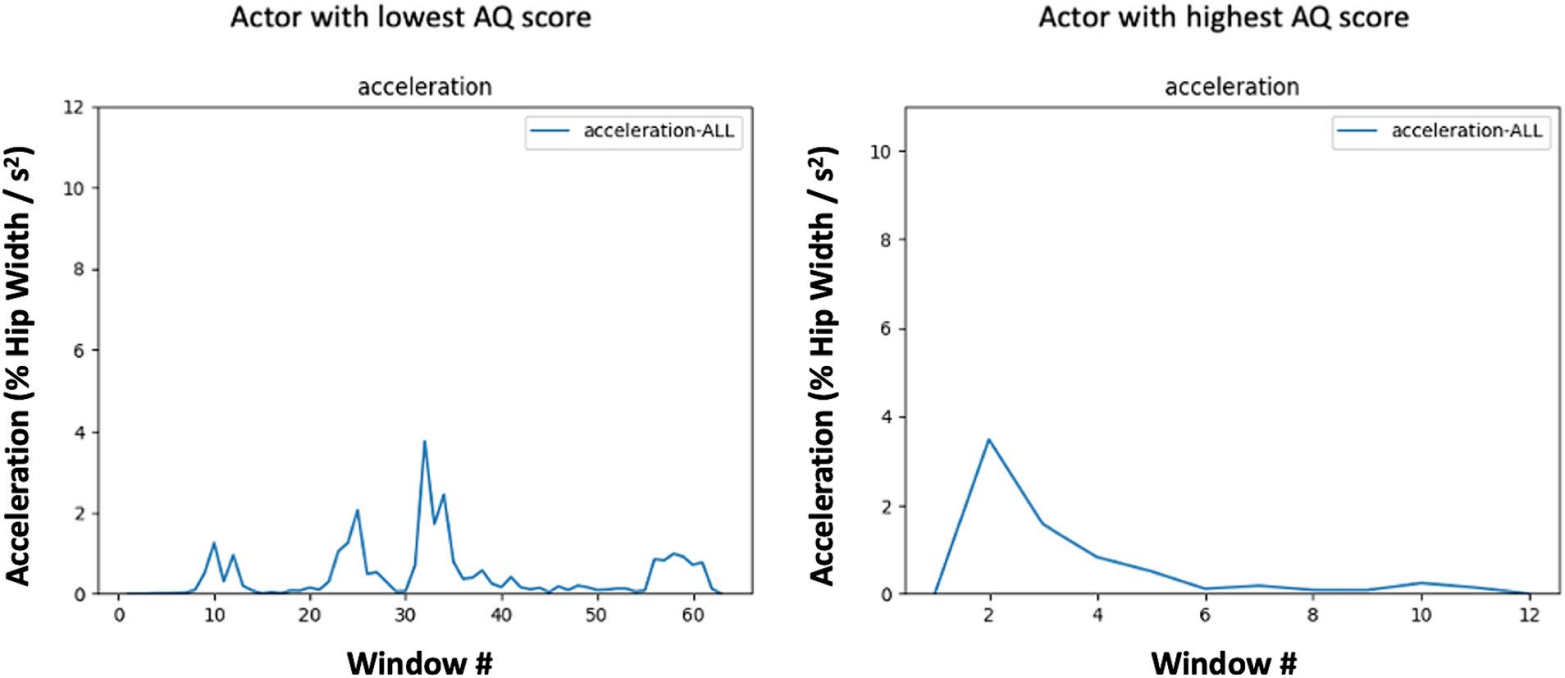
Mean absolute value of acceleration in each 7-frame window across the expression of anger by subjects with low (left) and high (right) AQ scores. Acceleration profiles are based on the center of mass of all fiducial points. Note the periodic nature of the low-AQ exemplar (left), which corresponds to a lower entropy level due to the repeated patterning of movement and the pulsatile and abrupt nature of the high-AQ exemplar’s (right) movement which demonstrates lower AUC for a similar level of Max acceleration.

Conversely, the greater regularity or structure of upper body movements might reflect subtle restricted and/or repetitive movement patterns often observed in individuals with higher autistic-like traits (Smith et al., 2009) contributing to predictability in their actions and consequently lower entropy in these regions (see Figure 3). Interestingly, these patterns of results resemble findings by Vabalas et al. in which motion tracking of simple pointing and aiming movements of the hand in individuals with and without ASD revealed slower, more restricted, and accurate movements in ASD compared to typically developing individuals (Vabalas et al., 2019). The notion of more restricted kinematics in the autism spectrum is also consistent with recent work by Zhao and colleagues (e.g., Zhao et al., 2021, 2022) in which entropy and amplitude was measured during arm oscillations in children with ASD and typically developing controls. Children with ASD showed lower entropy values, indicating more restricted movements. One interpretation of Zhao et al.’s finding is that children with ASD may deviate less from the preferred movement, resulting in less complexity over time (Zhao et al., 2021). This reduction in the complexity of body movement also extends to face-to-face interactions with an experimenter (Zhao et al., 2022). Although more research is needed, it is possible that such movement characteristics may extend to neurotypical individuals with elevated autistic-like traits. An intriguing observation from our findings was that individuals with elevated *Communication* subscale scores tended to exhibit upper body movements with increased structure and predictability (lower entropy). This relationship may suggest that in social interactions, where nonverbal cues are pivotal, those with elevated autistic traits may rely more on deliberate movements to convey their intent.

The augmented volume and magnitude of upper body movements observed in our study also aligns with this notion of repetitive movement patterns often noted in autism research (Mahone et al., 2004). This increase could also indicate underlying motor planning and control nuances, mirroring challenges identified in previous kinematic studies. Building on the insights from Granner-Shuman et al. which correlate higher levels of autistic traits with a reduced ability to synchronize movements and faster initiation of motor plans (Granner-Shuman et al., 2021), we might interpret the increased volume (AUC) and magnitude (MAX) of upper body movements in our study as related phenomena. The elevated movement volume and intensity could reflect a divergent motor strategy among neurotypical individuals with higher levels of autistic-like traits, to achieve clearer communication through more pronounced physical gestures. The enhanced magnitude could also be a kinematic reflection of the faster movement execution observed by Granner-Shuman et al. (2021).

Our digital kinematics represent a subset of the available metrics that can be quantified from human movements. We selected these metrics based on their salience in the visual perception of movement and their suitability to analysis from the PLD data structure. Multiple dimensions of movement are captured across even these three metrics, and they are not strictly independent. For instance, the duration of the movement influences both the entropy (by permitting greater repetition of movement patterns) and AUC. Volume of movement as captured by AUC is also influenced by the MAX acceleration during the expression. Due to this covariation of movement features, we elected to explore each digital kinematic in its own model. Future extensions of this research must tackle the challenge of interpreting nuanced social movements without imposing this type of discretization. Human perception of movement is sensitive to each of these (and many more) dimensions of motion, and thus future work will integrate multiple kinematic dimensions in a multivariate analysis. At this early phase of exploratory analysis, these more complex models would have limited the interpretability of these results. A goal moving forward is to build visualization and interpretation tools to assist the autism research community in making use of these more nuanced results.

This study adds several additional insights. First, the results demonstrate that autistic traits are associated with distinctive, non-interactive movement patterns. That is, these traits can influence motor behavior independent of social engagement or interaction. In addition, consistent with previous research, the results show that the expression of these emotions is characterized not only by distinctive arm movements but also by head movements, in line with the idea that upper body regions play integral roles in the kinesthetic expression of emotions (De Meijer, 1989; Wallbott, 1998; Sawada et al., 2003). Finally, emotional expressions are vital communicative signals that transmit essential information about threats, social hierarchy, and individual states (Shariff and Tracy, 2011). Our study revealed that the kinematic properties of movements associated with expressing anger, fear, and happiness can be modulated by autistic traits, underscoring the impact of autistic traits on how emotions are physically manifested and highlight the need for further research into a wider array of emotional expressions and scenarios to uncover potential variations in how these traits interact.

One noteworthy limitation of this study, however, is that the sample acquired was predominantly female. Existing research highlights significant differences between males and females in the heritability and manifestation of autism (see Hull et al., 2017; Ferri et al., 2018 for reviews). Despite autism diagnoses being more common in males (Baron-Cohen et al., 2011; Ferri et al., 2018), there are studies that indicate that females with ASD often display more pronounced social impairments (Kirkovski et al., 2013; Evans et al., 2019). Given these facts, we controlled for possible sex differences in the linear mixed-effects models and found that across all models, the relationship between autistic-like traits and kinematic measures did not vary as a function of male versus female. Nonetheless, given the 80:20 female: male ratio in our study, future work should examine whether these effects replicate in samples with more male participants.

In conclusion, this study marks the beginning of a research trajectory into the kinematic markers of the broader autism phenotype (BAP). Our initial findings offer a proof of concept for the application of digitized kinematics, captured by cameras, and analyzed via computer vision, as a powerful method for identifying movement-based endophenotypic features of autism. Notably, our results present an initial understanding of how autistic traits are associated with specific motor patterns—increased movement and structure in the upper body and heightened entropy in lower extremities—as they relate to expression of emotions. This work illuminates the potential for deploying computer vision tools for two reasons: first, the data reduction possible for storing PLDs or their coordinate-based representations on the 2D plane of the camera are considerable, and second, reduction of human movement video to PLDs conceals personal identifiers while preserving relationship to the state of the individual nervous system. Future extension of this research will leverage these advantages to build a representative dataset of a broad sample of the human population to further refine models of the broader phenotype and its relation to movement. While mixed effects regression models afford a degree of interpretability that supports mechanistic investigations, as in this current work, subsequent phases will utilize machine learning methods to fully harness the nested nature of social movements. Digital kinematics, validated in their association with BAP through our work, stand as promising candidates for training these sophisticated models, with potential to significantly advance the phenotypic characterization of autism.

## Data availability statement

The datasets presented in this article are not readily available because of ongoing analyses and potential future publications based on additional aspects of the dataset. We are committed to ensuring the responsible and comprehensive use of the data, and access may be considered on a case-by-case basis. Researchers interested in accessing the dataset for collaboration or specific inquiries may contact the corresponding author to discuss potential arrangements and terms. Requests to access the datasets should be directed to MJ, mjaime@iu.edu.

## Ethics statement

The studies involving humans were approved by Human Subjects and Institutional Review Boards of Indiana University. The studies were conducted in accordance with the local legislation and institutional requirements. The participants provided their written informed consent to participate in this study.

## Author contributions

GL: Formal analysis, Resources, Writing – review & editing, Methodology, Software, Visualization. ES: Data curation, Funding acquisition, Investigation, Writing – original draft, Writing – review & editing. MA: Formal analysis, Writing – review & editing, Software. SD: Writing – original draft, Writing – review & editing. MJ: Conceptualization, Data curation, Formal analysis, Funding acquisition, Investigation, Methodology, Project administration, Resources, Supervision, Writing – original draft, Visualization, Writing – review & editing.
